# NADPH Oxidase Deficient Mice Develop Colitis and Bacteremia upon Infection with Normally Avirulent, TTSS-1- and TTSS-2-Deficient *Salmonella* Typhimurium

**DOI:** 10.1371/journal.pone.0077204

**Published:** 2013-10-15

**Authors:** Boas Felmy, Pascal Songhet, Emma Marie Caroline Slack, Andreas J. Müller, Marcus Kremer, Laurye Van Maele, Delphine Cayet, Mathias Heikenwalder, Jean-Claude Sirard, Wolf-Dietrich Hardt

**Affiliations:** 1 Institute of Microbiology, D-BIOL, ETH Zürich, Zurich, Switzerland; 2 Institut für Allgemeine Pathologie und Pathologische Anatomie, Technische Universität München, Munich, Germany; 3 Institut Pasteur de Lille, Center for Infection and Immunity of Lille; Institut National de la Santé et de la Recherche Médicale; CNRS, UMR 8204; University Lille Nord de France, Lille, France; 4 Institute for Virology, Technical University Munich/Helmholtz Center Munich, Munich, Germany; KIIT University, India

## Abstract

Infections, microbe sampling and occasional leakage of commensal microbiota and their products across the intestinal epithelial cell layer represent a permanent challenge to the intestinal immune system. The production of reactive oxygen species by NADPH oxidase is thought to be a key element of defense. Patients suffering from chronic granulomatous disease are deficient in one of the subunits of NADPH oxidase. They display a high incidence of Crohn’s disease-like intestinal inflammation and are hyper-susceptible to infection with fungi and bacteria, including a 10-fold increased risk of Salmonellosis. It is not completely understood which steps of the infection process are affected by the NADPH oxidase deficiency. We employed a mouse model for *Salmonella* diarrhea to study how NADPH oxidase deficiency (*Cybb* ^−/−^) affects microbe handling by the large intestinal mucosa. In this animal model, wild type *S*. Typhimurium causes pronounced enteropathy in wild type mice. In contrast, an avirulent *S*. Typhimurium mutant (*S*.Tm^avir^; *invGsseD*), which lacks virulence factors boosting trans-epithelial penetration and growth in the lamina propria, cannot cause enteropathy in wild type mice. We found that *Cybb* ^−/−^ mice are efficiently infected by *S*.Tm^avir^ and develop enteropathy by day 4 post infection. Cell depletion experiments and infections in *Cybb* ^−/−^ *Myd88* ^−/−^ mice indicated that the *S*.Tm^avir^-inflicted disease in *Cybb* ^−/−^ mice hinges on CD11c^+^CX_3_CR1^+^ monocytic phagocytes mediating colonization of the cecal lamina propria and on *Myd88*-dependent proinflammatory immune responses. Interestingly, in mixed bone marrow chimeras a partial reconstitution of C*ybb*-proficiency in the bone marrow derived compartment was sufficient to ameliorate disease severity. Our data indicate that NADPH oxidase expression is of key importance for restricting the growth of *S*.Tm^avir^ in the mucosal lamina propria. This provides important insights into microbe handling by the large intestinal mucosa and the role of NADPH oxidase in maintaining microbe-host mutualism at this exposed body surface.

## Introduction

The intestinal immune system is capable of handling occasional breaches by the microbiota and by mucosal-invading pathogens. This is facilitated by efficient secondary barriers, such as the large number of specialized lymphoid and myeloid cells of the gut-associated immune system (e.g. Peyer’s patches and isolated lymphoid follicles) and the lamina propria (LP) of the absorptive mucosa. Normally, commensals and pathogens which breach the epithelial layer are taken up, killed, processed and presented by diverse phagocytes, in particular by diverse mononuclear phagocyte populations and polymorphonuclear leukocytes/granulocytes (PMN). Therefore, these populations are thought to play an important role in limiting bacterial loads in the LP and preventing disease.

In the infected mucosa, a mixture of different phagocytes is found. This includes the PMN and at least three different monocytic phagocyte populations, i.e. dendritic cells performing functions in antigen transport and presentation (e.g. CD11b^+^CD11c^+^CD103^+^CX_3_CR1^−^ cells), macrophages contributing to microbe phagocytosis and elimination (e.g. CD11b^+^CD11c^−^CD103^−^CX_3_CR1^−^ cells) and CX_3_CR1^+^ mononuclear phagocytes (e.g. CD11b^+^CD11c^+/−^CD103^−^CX_3_CR1^+^ cells) which are thought to facilitate luminal antigen sampling, eliciting T_H_1 and T_H_17 differentiation, and to control pro- and anti-inflammatory responses [1].

The antimicrobial repertoire of PMN includes proteases and reactive oxygen species (ROS) produced by the NADPH oxidase complex, containing CYBB [2]. Interestingly, NADPH oxidase deficiency leads to a pronounced susceptibility to bacterial infection and inflammatory disease [3], [4]. This condition is termed chronic granulomatous disease (CGD) and is traceable to genetic disruptions of NADPH oxidase, i.e. in approximately 65% of cases to mutations of the *Cybb* gene encoding the cytochrome b-245 H chain catalytic subunit [5]. CGD patients are highly susceptible to systemic infection and/or granuloma formation by *Staphylococcus* spp., *Mycobacterium* spp., *Salmonella* spp., *Aspergillus* spp., *Pseudomonas* spp. and *Burkholderia cepacia* and chronic gut inflammation resembling inflammatory bowel diseases [5]–[7]. The latter indicates that NADPH oxidase is of significant importance for limiting microbe growth and/or access to the LP and/or regulation of inflammation in the intestine [8], [9].

To analyze NADPH oxidase mediated defense in the intestinal mucosa, we have employed a mouse model for *Salmonella enterica* subspecies 1 serovar Typhimurium (*S*. Typhimurium) diarrhea. CGD patients display an approximately 10-fold increased rate of infection with *Salmonella* spp. than the normal population and *Salmonella* spp. have been isolated from stools of CGD patients with intestinal inflammation [6], [7], [10]. Similarly, *S*. Typhimurium grows in systemic sites in NADPH oxidase deficient and in PMN-depleted mice [3], [11]–[17]. However, the importance of *Cybb* expression by PMN in preventing mucosal infection has not been fully understood.

Two different versions of the streptomycin pretreated mouse model for *S*. Typhimurium diarrhea [18] were of particular interest for probing NADPH oxidase function in the gut. In the standard model [19], mice are infected with wild type *S*. Typhimurium and develop a pronounced gut inflammation in the cecum. In contrast, isogenic *S*. Typhimurium mutants lacking type three secretion system (TTSS)-1 and TTSS-2, responsible for the secretion of virulence factors boosting epithelial cell invasion and pathogen growth within LP phagocytes, do not cause disease. In a second version of this model, which employs *S*. Typhimurium mutants lacking a functional TTSS-1 (e.g. SL1344 Δ*invG*, *S*. Tm*^invG^*), the pathogen relies on CD11c^+^CX_3_CR1^+^ monocytic phagocytes to traverse the epithelial barrier, grows within CD11c^−^CX_3_CR1^−^ monocytes of the LP and causes overt mucosal inflammation 3 days post infection (p.i.) [20], [21]. This model allows analysis of pathogen virulence factors (e.g. TTSS-2; [20]) as well as the mechanisms used by the host to restrict pathogen growth within mucosal monocytic phagocytes [18].

Using these well-established mouse models for *S*. Typhimurium colitis, we have analyzed the role of NADPH oxidase in the infected mucosa. Our findings might be of general importance for understanding pathogen and commensal handling by the mucosal immune system and might help to understand the effects of a partial restoration of *Cybb*-functionality in CGD patients by gene therapy or bone marrow transfer.

## Results

### *Cybb^−/−^* Mice Fail to Control Infection with a Normally Avirulent *S*. Typhimurium Mutant

To analyze the role of NADPH oxidase in mucosal defense, we have worked in the genetic background of *S*. Tm*^invG^* (lacking TTSS-1). *S*. Tm*^invG^* requires CD11c^+^CX_3_CR1^+^ monocytic phagocytes for traversing the epithelial barrier, grows within the LP and elicits enteropathy in a *Myd88*-dependent fashion by day 3 p.i. in wild type mice. This has been termed the “alternative pathway” [18], [21]. We speculated that this pathway might be particularly sensitive for NADPH oxidase deficiency, as *Cybb* might help restricting bacterial growth in the LP.

**Figure 1:**
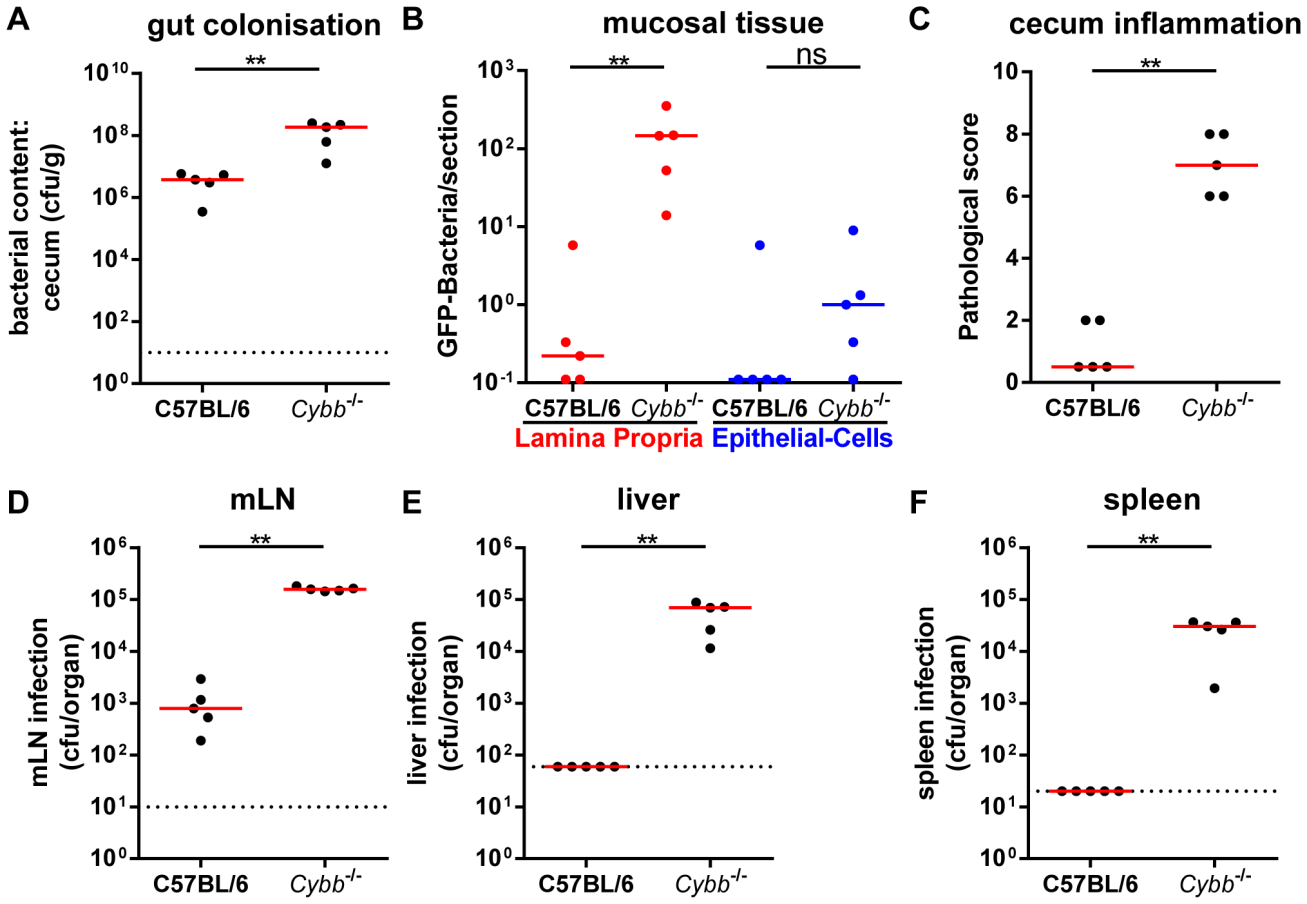
*S*.Tm^avir^ infection of *Cybb^−/−^* mice leads to pathogen growth in the LP and enteropathy. *Cybb* ^−/−^ mice (C57BL/6 background) and C57BL/6 control mice were pretreated with streptomycin and infected for 4 days with *S*.Tm^avir^. The bacterial loads in the gut lumen (A), the LP (red (B)) or the epithelial cells of the cecum (blue (B)), the degree of mucosal inflammation (C) and bacterial loads in the mLNs (D), livers (E) and spleens (F) were analyzed. *: p<0.05; **: p<0.01; ns: not significant; red line: median; dashed line: minimal detectable value.

We pretreated wild type and *Cybb* ^−/−^ mice with streptomycin and infected them for 4 days with *S*.Tm^avir^ (5×10^7^ cfu by gavage) to analyze the role of *Cybb* in restricting the growth of *S*.Tm^avir^ in the mucosal tissue. High *S*.Tm^avir^ loads were detected in the gut lumen of wild type and *Cybb* ^−/−^ mice (Fig. 1A). Bacterial loads in the LP were significantly lower in the wild type than in the *Cybb* ^−/−^ animals (Fig. 1B) and only the latter developed pronounced mucosal inflammation by day 4 p.i. (Fig. 1C). Furthermore, the *Cybb* ^−/−^ mice displayed significantly increased loads of *S*.Tm^avir^ in the mesenteric lymph nodes (mLNs, Fig. 1D), the livers (Fig. 1E) and the spleens (Fig. 1F) compared to C57BL/6 mice. This high susceptibility to systemic spread was expected as NADPH oxidase is known to be of key importance for limiting systemic infections [3], [4]. Our data extended these findings by showing that NADPH oxidase is essential for restricting the growth of *S*.Tm^avir^ not only at systemic sites, but also in the cecal LP.

### iNOS does not Contribute Significantly to Mucosal Defense against *S*.Tm^avir^

The inducible NO synthase (iNOS) is an important defense mechanism of monocytic macrophages [2] and can help restricting pathogen growth in various models [22]–[27]. In order to assess the role of iNOS in our infection model, we included iNOS-deficient (*Nos2^−/−^*) animals and *Cybb^−/−^Nos2^−/−^* double KO mice into the infection experiments with *S*.Tm^avir^ shown in Fig. 1. Neither cecum pathology (Fig. S1C) nor tissue loads (Fig. S1B, D–F) in the *Nos2^−/−^* mice differed from wild type C57BL/6 animals. Similarly, the cecum pathology (Fig. S1C) and the tissue loads in the cecal mucosa (Fig. S1B) and the livers (Fig. S1E) did not differ significantly between the *Cybb^−/−^* and the *Cybb^−/−^Nos2^−/−^* mice. The *Cybb^−/−^Nos2^−/−^* animals displayed slightly but significantly elevated *S*.Tm^avir^ tissue loads only in the mLNs (Fig. S1D) and the spleens (Fig. S1F). However, even in these organs, *Cybb*-deficiency had a more pronounced effect than *Nos2*-deficiency and significant contributions of *Nos2* were only detectable in the presence of *Cybb*, suggesting a possible synergistic role for *Nos2* [16]. In conclusion, restriction of *S*.Tm^avir^ in the cecal mucosa and the protection from enteropathy seems to hinge on NADPH oxidase while iNOS seems to contribute little (maximally in a synergistic manner) to mucosal defense, at least during the first 4 days of infection.

### Increased Mucosal NADPH Oxidase Expression in Response to Infection of Wild Type Mice with Wild Type *S*. Typhimurium

The standard streptomycin model for murine *S*. Typhimurium diarrhea was used to assess *Cybb* expression in the infected mucosa of wild type C57BL/6 mice. Streptomycin pretreated animals were infected with wild type *S*. Typhimurium (*S*.Tm^wt^; 5×10^7^ cfu by gavage) for 12 or 24 h. Samples of the cecum tissue (the site of the initial and most pronounced enteropathy [18], [19]) were recovered to analyze *Cybb* expression by reverse transcription quantitative real-time PCR (RT-qPCR). In line with earlier data [28], the abundance of *Cybb* mRNA in the cecum increased by about 3-fold after 12 h and about 8-fold after 24 h of infection compared to streptomycin-treated animals (Fig. S2A). This went along with mucosal inflammation and infiltration of neutrophils and monocytic phagocytes into the cecal mucosa as observed by histopathology and flow cytometry analysis (Fig. S2B, C, D).

### *S*.Tm^avir^ Colitis in *Cybb* ^−/−^ Mice is Similarly Dependent on *Myd88* and Mucosal CD11c^+^ Monocytic Phagocytes as *S*.Tm*^invG^*–induced Colitis in Wild Type C57BL/6 Mice

The role of *Cybb* and or PMN in the acute infection is not completely understood. Thus, we performed a number of control experiments to analyze the pathogenetic mechanism of *S*.Tm^avir^ colitis in *Cybb* ^−/−^ mice. First, we analyzed uninfected and infected gut tissues with immuno-histopathological stainings for markers characteristic for a set of immune cells. The *S*.Tm^avir^ infected mucosa of *Cybb* ^−/−^ mice at day 4 p.i. displayed a patchy pathology characterized by non-inflamed regions interspaced with pronounced inflammatory foci (Fig. S3). Such patchy pathology is characteristic for *S*.Tm*^invG^* (lacking only TTSS-1) infections at day 3 p.i. in wild type C57BL/6 mice (Fig. S4; [20]). This provided first hints suggesting that *Cybb*-deficiency allows *S*.Tm^avir^ to elicit enteropathy via the “alternative pathway”. In line with this, RT-qPCR analysis of a panel of 27 genes for cytokines or antimicrobial defenses revealed similar mucosal gene expression profiles (Fig. S4G) in *Cybb* ^−/−^ mice (4 days p.i. with *S*.Tm^avir^) and wild type C57BL/6 mice (3 days p.i. with *S*.Tm*^invG^*) at their first day of overt enteropathy (Fig. S4). In this experiment, the counts in the mLNs (Fig. S4D), livers (Fig. S4E) and spleens (Fig. S4F) were lower in the C57BL/6 mice compared to *Cybb^−/−^* mice. However, most importantly and in line with the RT-qPCR data, the degree of cecum inflammation was alike (Fig. S4B, C).

**Figure 2:**
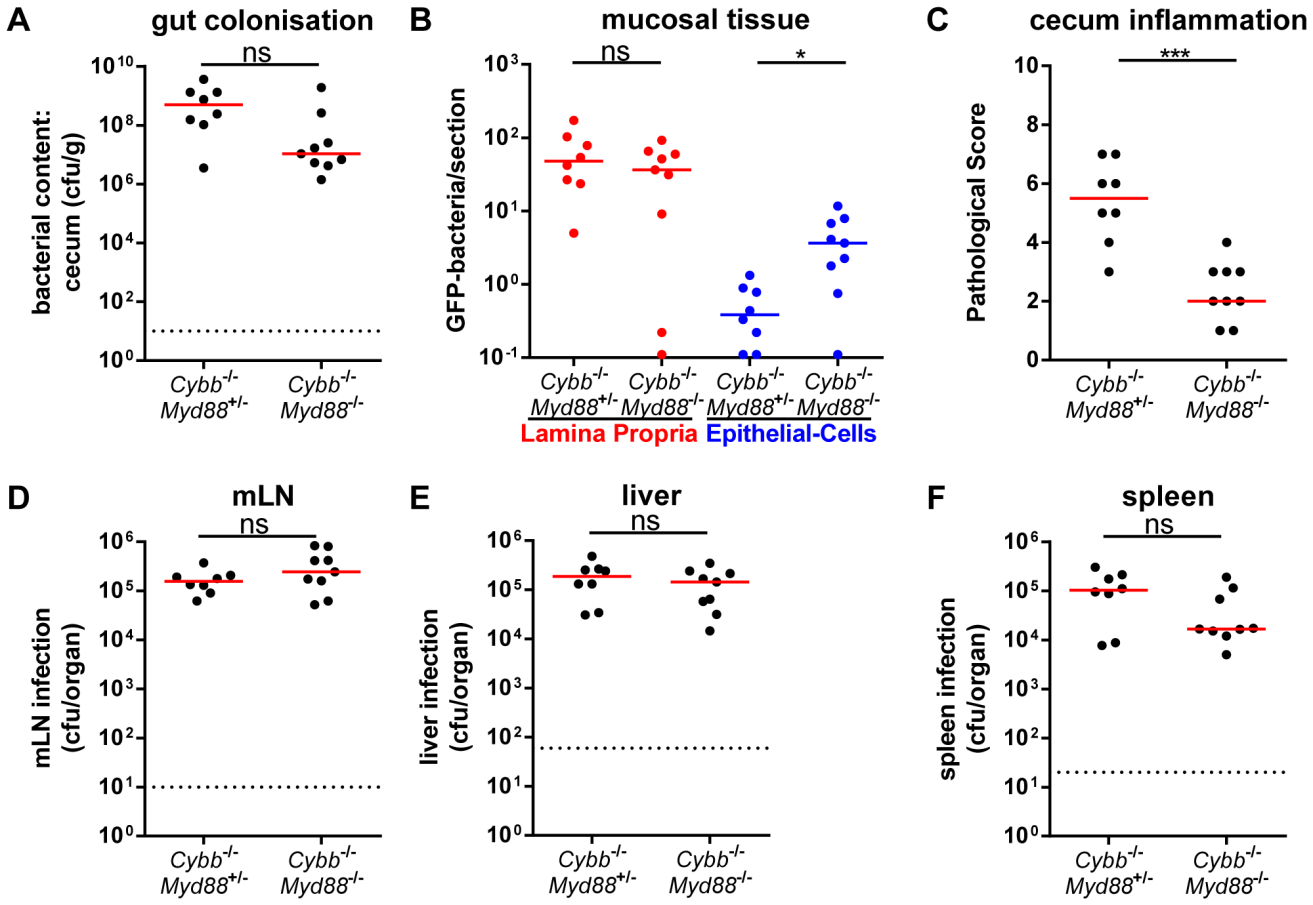
*Myd88*-dependency of enteropathy in *S*.Tm^avir^ infected *Cybb* ^−/−^ mice. *Cybb* ^−/−^ *Myd88* ^+/−^ mice or C*ybb* ^−/−^ *Myd88* ^−/−^ littermates mice were pretreated with streptomycin and infected with *S*.Tm^avir^ for 4 days. The bacterial loads in the gut lumen (A), the LP (red (B)) or the epithelial cells of the cecum (blue (B)), the degree of mucosal inflammation (C) and bacterial loads in the mLNs (D), livers (E) and spleens (F) were analyzed. *: p<0.05; **: p<0.01; ns: not significant; red line: median; dashed line: minimal detectable value.

We then assessed the *Myd88*-dependency of the inflammatory response as a typical feature of the “alternative pathway”. For this purpose we infected *Cybb* ^−/−^ *Myd88* ^−/−^ mice or *Cybb* ^−/−^ *Myd88* ^+/−^ littermate controls for 4 days with *S*.Tm^avir^ (5×10^7^ cfu by gavage). High loads of *S*.Tm^avir^ were observed in the gut lumen (Fig. 2A), LP (Fig. 2B), mLNs (Fig. 2D), livers (Fig. 2E) and spleens (Fig. 2F) of both groups. However, only the *Myd88*-proficient mice developed mucosal inflammation, while the *Cybb* ^−/−^ *Myd88* ^−/−^ animals did not (Fig. 2C). It is interesting to note that the *Cybb* ^−/−^ *Myd88* ^−/−^ animals displayed slightly but significantly elevated *S*.Tm^avir^ loads in the cecal epithelium (Fig. 2B; blue symbols). It is unclear whether this might be explained by reduced epithelial turnover rates of non-infected tissue in *Myd88* ^−/−^ animals [29]. Alternatively, this might be indicative of a *Myd88*-dependent, but *Cybb*-independent defense mechanism which may contribute to limiting bacterial growth in the enterocytes. Such mechanisms could be an interesting topic for future research. To this end, the data verified the *Myd88*-dependency of enteropathy in *S*.Tm^avir^ infected *Cybb* ^−/−^ mice.

**Figure 3:**
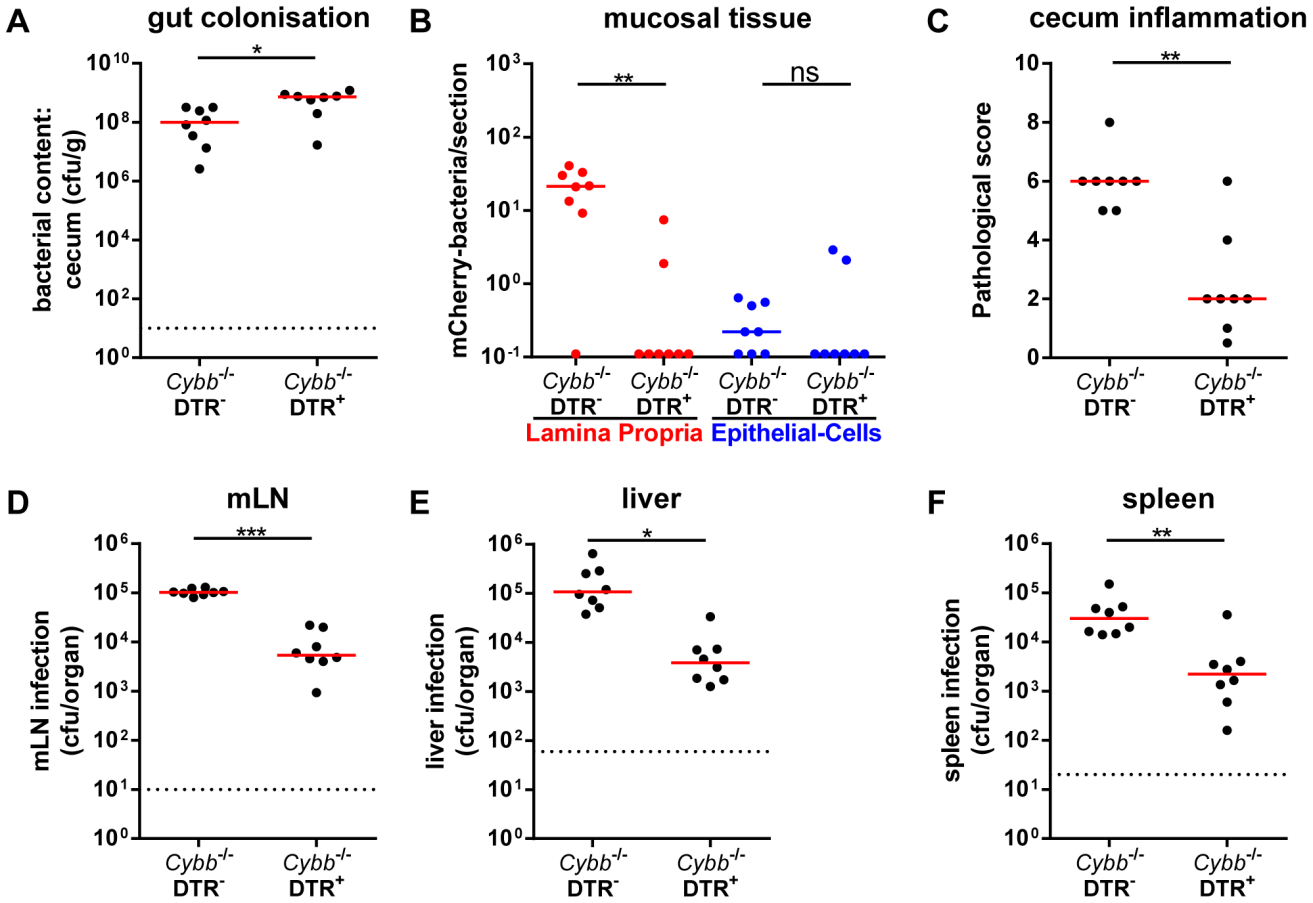
Cell depletion demonstrating the monocytic phagocyte dependency of *S*.Tm^avir^ infection in *Cybb* ^−/−/^DTR^+^ mice. *Cybb* ^−/−/^DTR^+^ mice and *Cybb* ^−/−/^DTR^−^ littermates were pretreated with streptomycin, injected with DTX 18 h prior to and 30 h post infection and infected for 4 days with *S*.Tm^avir^. The bacterial loads in the gut lumen (A), the LP (red (B)) or the epithelial cells of the cecum (blue (B)), the degree of mucosal inflammation (C) and bacterial loads in the mLNs (D), livers (E) and spleens (F) were analyzed. *: p<0.05; **: p<0.01; ns: not significant; red line: median; dashed line: minimal detectable value.

Finally, we have assessed the dependency on mucosal monocytic phagocytes. Using transgenic mice expressing the diphtheria-toxin receptor under control of the CD11c promoter (DTR^+/−^; [30]) and diphtheria toxin-mediated (DTX) cell depletion, it has been previously established that *S*.Tm*^invG^* relies on mucosal CD11c^+^ monocytic phagocytes for traversing the gut epithelium and colonizing the cecal LP [21]. Thus, *Cybb* ^−/−^DTR^+/−^ mice or *Cybb* ^−/−^ littermates were treated with DTX and infected for 4 days with *S*.Tm^avir^. High loads of *S*.Tm^avir^ were detected in the gut lumen of both groups (Fig. 3A). In contrast, the DTX-mediated cell depletion abolished mucosa tissue infection (Fig. 3B) and the elicitation of mucosal inflammation (Fig. 3C). Furthermore, it significantly reduced the infection of mLNs (Fig. 3D), livers (Fig. 3E) and spleens (Fig. 3F). These data were all in line with the notion that *S*.Tm^avir^ infection of *Cybb* ^−/−^ mice follows a similar pathogenetic mechanism as described earlier for the *S*.Tm*^invG^* infection in wild type mice. The key difference between both infections seems to reside in the failure of the *Cybb* ^−/−^ mice to control *S*.Tm^avir^ infection/growth in the infected mucosa. This would suffice to explain the susceptibility of *Cybb* ^−/−^ (but not wild type) mice to *S*.Tm^avir^-triggered enteropathy and suggests that the model might be of interest for studying the role of *Cybb* in restricting bacterial growth in the cecal LP.

### *S*.Tm^avir^ Induces Intermediate Levels of Enteropathy in Mice Reconstituted with a Mix of *Cybb* ^−/−^ and Wild Type Bone Marrow

**Figure 4:**
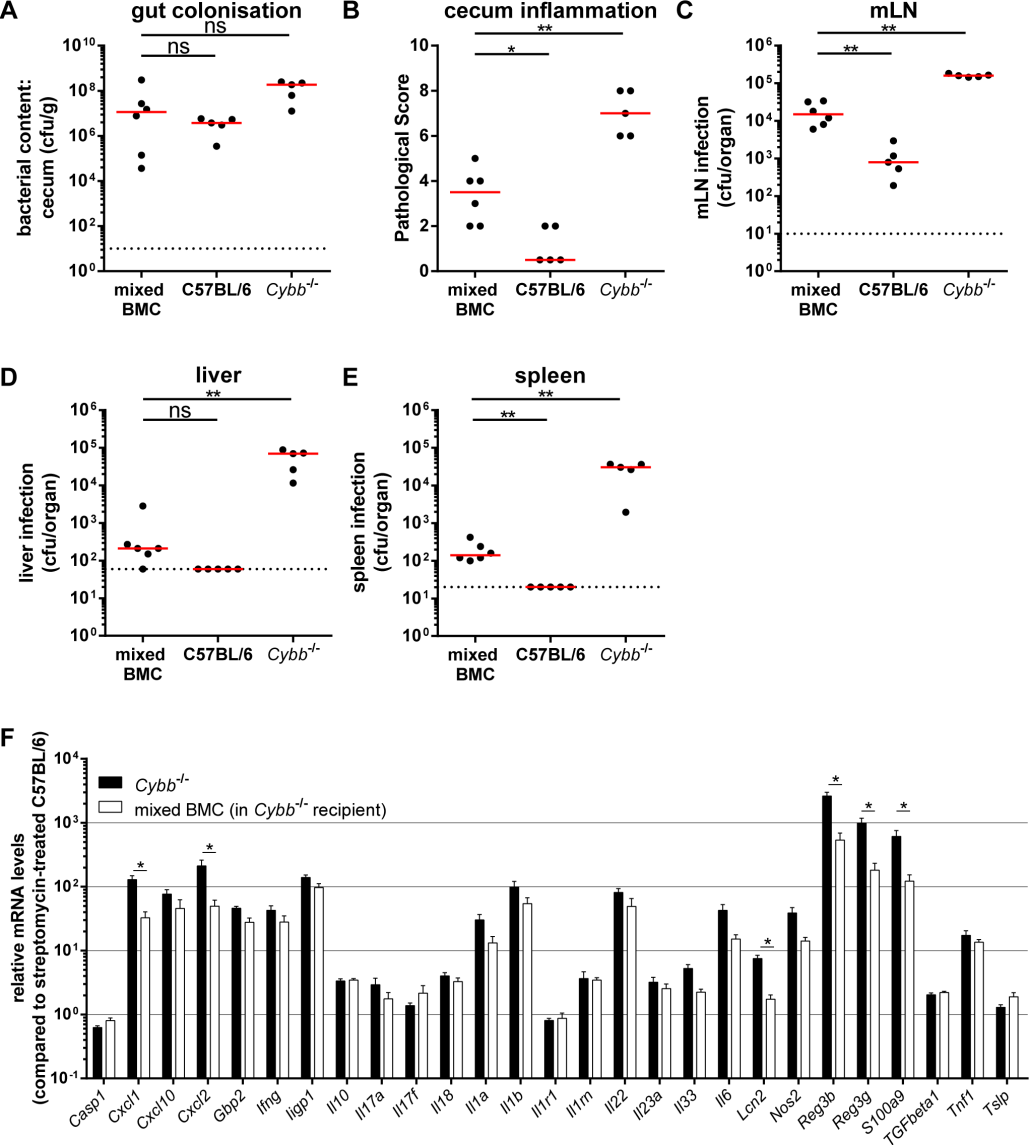
31% wild type cells in *Cybb^−/−^*mice are sufficient to reduce systemic loads of *S*.Tm^avir^. *Cybb* ^−/−^ mice were irradiated and reconstituted with a mix of C57BL/6 Ly5.1 and *Cybb* ^−/−^ Ly5.2 bone marrow (mixed BMC). Mice were pretreated with streptomycin and infected for 4 days with *S*.Tm^avir^. The bacterial loads in the gut lumen (A), the degree of mucosal inflammation (B) and bacterial loads in the mLNs (C), livers (D) and spleens (E) were analyzed. The data for the C57BL/6 and *Cybb* ^−/−^ mice were replotted from Fig. 2 for a better comparison. Relative mRNA expression levels were compared between bone marrow chimeras and similarly treated *Cybb* ^−/−^ mice, data partly replotted from Fig. S4G for a better comparison (F). Data is displayed as mean + SEM (F); *: p<0.05; **: p<0.01; ns: not significant; red line: median; dashed line: minimal detectable value.

To further analyze how *Cybb* restricts *S*.Tm^avir^ infection of the cecal mucosa, we performed an experiment with mixed bone marrow chimeric mice. The chimeras were generated by reconstituting irradiated *Cybb* ^−/−^ mice (congenic marker Ly5.2) with a mix of 50% *Cybb* ^−/−^ (congenic marker Ly5.2) and 50% C57BL/6 (congenic marker Ly5.1) bone marrow. After 8 weeks, these chimeras displayed 69% *Cybb* ^−/−^ (congenic marker Ly5.2) and 31% C57BL/6 (congenic marker Ly5.1) cells in the cecal LP as tested by flow cytometry at the end of the experiment (data not shown). In these mice, the stromal cells and all CD45.2^+^ cells (i.e. phagocytes, B-cells, T-cells, etc.) were *Cybb*-deficient, while the CD45.1^+^ cells were *Cybb*-proficient. Four days p.i. with *S*.Tm^avir^ all chimeric mice (mixed BMC, Fig. 4) displayed high pathogen loads in the gut lumen (Fig. 4A) and significant amounts of bacteria in the mLNs (Fig. 4C) and spleens (Fig. 4E). The levels of mLN and spleen colonization in the mixed chimeras were lower than in the *Cybb* ^−/−^ mice, but higher than in the wild type C57BL/6 animals (Fig. 4C, E). The same intermediate phenotype was observed for the cecum pathology (Fig. 4B), whereas liver colonization was not distinguishable from wild type C57BL/6 animals (Fig. 4D). Interestingly, approximately one third of *Cybb*-proficient cells in a *Cybb*-knock out background lead to a reduction of bacterial counts in spleens and livers by 217- and 333-fold (ratio between the medians), respectively, if compared with mice completely deficient for *Cybb*. Additionally, RT-qPCR analysis of proinflammatory cytokines confirms the alleviated inflammatory phenotype (Fig. 4F). These data indicate that *Cybb*-proficiency in only 31% of the bone-marrow-derived compartment is sufficient to achieve a significant restriction of *S*.Tm^avir^ colonization of the host tissue and enteropathy.

## Discussion

Here, we have analyzed NADPH oxidase defenses of the intestinal mucosa. We established that NADPH oxidase deficient mice were not able to limit gut mucosa colonization and enteropathy by a normally avirulent *S*. Typhimurium strain. This demonstrated that disease via the “alternative” pathway hinges on a fine balance between microbe entry into the LP, microbe growth at this site and pathogen killing in the LP. Our data confirmed that LP access is controlled at least in part by dendritic cells (monocytic phagocytes), and demonstrated that microbe growth/killing is controlled by bacterial virulence factors (e.g. TTSS-2) and host defenses (e.g. NADPH oxidase-mediated killing in PMN).

While the central role of NADPH oxidase, i.e. CYBB, is well established in antimicrobial defense, the nature of the cell types facilitating the NADPH oxidase dependent defenses had remained less clear. The role of NADPH oxidase in the anti-microbial activity of neutrophils is well established [21], the same holds true for its role in dendritic cell-mediated antigen presentation and T-cell priming [31]–[33]. However, what is the mechanism activating NADPH oxidase in the mucosal phagocytes? Besides NADPH oxidase-deficiency/CGD, other primary immune deficiencies enhancing susceptibility to bacterial infection are deficiencies in Toll-like receptor- and IFNγ-R-signalling [34], [35]. In mouse models for systemic and intestinal *Salmonella* infection, Toll-like receptor - and IFNγ-R-signalling were indeed found to restrict pathogen growth [20], [36]–[42]. NADPH oxidase (and iNOS) are known to be activated via both MyD88- and IFNγ-signalling. However, *S*.Tm^avir^ did not colonize the LP of MyD88^−/−^ or IFNγ-R^−/−^ mice and did not cause enteropathy (this work and data not shown). This indicated that NADPH oxidase is activated by several redundant signalling pathways in LP cells. Deciphering such signalling pathways and the cell type mainly responsible for NADPH oxidase expression will be an interesting topic for future work. The *S*.Tm^avir^ infection model would be well suited for such studies, because *S*. Tm^avir^ offers well defined genetics and virulence factors. The removal of the latter from *S*. Tm^wt^ still leads to disease in a mouse model of CGD. This indicates that even very low virulence is sufficient to cause enteropathy in mice deficient in a subunit of the NADPH oxidase, broadening our understanding of how commensals might induce enteropathy in CGD patients.

The diffusible nature of some of the ROS (i.e. hydrogen peroxide) has raised some interest, as neighboring cells might be affected, even if they are not by themself capable of expressing NADPH oxidase. This has in fact been demonstrated *in vitro* [43], [44] and has thus complicated the interpretation of data from mouse experiments with cell-type specific NADPH oxidase deficiencies [45].

Our data demonstrate that the augmentation by neighboring cells (by ROS-signalling or by wild type cell mediated decreases of the pathogen loads) might be indeed of importance, at least in the *S*.Tm^avir^ infected intestinal mucosa. The mixed *Cybb*-proficient and -deficient bone marrow chimeras displayed >200× lower systemic *S*.Tm^avir^ loads than the *Cybb* ^−/−^ controls. Apparently, 31% of *Cybb*-proficient CD45^+^ cells are sufficient for this. This is in line with other publications focusing on *A. fumigatus* infections. *In vitro*, *A. fumigatus* hyphae could be damaged by a mixture of normal and “CGD neutrophils” [46]. Furthermore, *Cybb* ^−/−^ mice with >92% *Cybb*-deficient and 4–8% *Cybb*-proficient cells were fully protected [46], [47] to challenge with a dose of *A. fumigatus* sufficient to cause disease in *Cybb* ^−/−^ mice. Furthermore, the reported amount of *Cybb*-proficient cells necessary to respond similarly to an infection (i.e. survive) as wild type mice is 21–35% or 32–41% for challenge with *S*. *aureus* or *B*. *cepacia*, respectively [47]. Similarly, survival of CGD patients after entering adulthood was strongly associated with residual reactive oxygen intermediates production [48]. In extension, our data and the evidence from the other infection models discussed above indicate that even a partial therapy of CGD patients might be sufficient to significantly decrease their disease susceptibility far beyond the degree of achieved reconstitution. The need for less than 100% reconstitution (as typically observed in gene therapy [49]) might be of relevance for preclinical testing and the design of gene therapy approaches for treating CGD.

Up to date, it is unclear why a partial restoration of *Cybb* expression is sufficient to ameliorate the phenotype drastically. There are three possible explanations.

Firstly, we showed recently that *S*. Tm*^invG^* is found exclusively in CD11c^+^ cells at 1 day p.i. in our infection mouse model and only from 2 days p.i. on also in CD11c^−^ cells [21], [50]. This mechanism might also apply for the *S*. Tm^avir^ infection mouse model, since the S. Tm*^invG^* infection in C57BL/6 mice seems to be phenotypically similar to S. Tm^avir^ infection in *Cybb* ^−/−^ mice. The transition between CD11c^+^ and CD11c^−^ cells can possibly be the reason for an incidental exposure (and killing) of some *S*. Tm^avir^ bacteria to *Cybb*-proficient phagocytes. Killing in the *Cybb*-proficient phagocyte populations could explain the reduced tissue loads and disease pathology of the mixed bone marrow chimeras. Secondly, ROS produced by *Cybb*-proficient cells play an important role in controlling signalling pathways. Here, the reversible oxidation and inactivation of protein tyrosine phosphatases and MAP kinase phosphatases by ROS are interesting examples [51]. As a measure for proinflammatory signalling levels, we quantified mRNA levels of 27 genes related to inflammation and defense against *S*. Typhimurium infection. However, although 6 out of 27 genes were expressed less in the *S*.Tm^avir^ infections of mixed bone marrow chimeras compared to *Cybb* ^−/−^deficient mice, 21 out of 27 gene expression levels were similarly induced, if induced at all. This might indicate that only a part of the signalling pathways are affected by *Cybb*-deficiency.

Thirdly, we cannot exclude that the, already discussed, diffusion of some of the ROS (i.e. hydrogen peroxide) from *Cybb*-proficient cells into neighboring *Cybb*-deficient cells described *in vitro* [43], [44] may also occur *in vivo*. The current data is insufficient to tease apart these three mechanistic explanations. Nonetheless, the reduced disease severity of *S*. Tm^avir^ infections in the mixed bone marrow chimeras provides a useful basis for addressing this issue.

In CGD patients, Crohn’s disease-like inflammation of the intestinal mucosa is frequently observed [7], [52]. *Salmonella* spp. can be isolated from the stools of some, but clearly not from all of these patients [7]. This indicates that, on the one hand, growth restriction of normally avirulent *Salmonella* by NADPH oxidase may be of relevance for CGD patients, but on the other hand that other microbial stimuli can also trigger enteropathy. In the “non-*Salmonella*-related” cases, inflammation might be attributable to insufficient restriction of commensal microbiota species which would not cause disease in NADPH oxidase proficient hosts. In this case, NADPH oxidase-mediated growth restriction by LP cells may function as an immunological barrier of general importance for maintaining homeostasis in the intestinal mucosa. Our findings might be of importance for understanding microbe handling by the intestinal immune system and for elucidating strategies employed by pathogens to overcome this defense.

Our findings may also contribute to our understanding of the evolution of *S*. Typhimurium as a successful enteropathogen. During its divergence from a commensal *E. coli* lineage, this pathogen has acquired two novel genetic loci of importance for enteropathogensis which encode the two TTSSs [53]–[55]. In wild type hosts, TTSS-2 was shown to enhance pathogen survival in LP phagocytes and thereby enhance mucosal inflammation [20], [21], [56]–[58]. Tissue culture experiments suggested that this is attributable to TTSS-2 dependent interference with NADPH oxidase (or iNOS-) delivery to the *Salmonella* containing phagosome [59], [60]. This is supported by our finding that *S*.Tm^avir^, which lacks a functional TTSS-2, is only capable of colonizing the LP and cause enteropathy in *Cybb*-deficient, but not in wild type mice. In conclusion, TTSS-2 may represent a pathogen-specific adaptation to overcome and subvert the NADPH oxidase mediated mucosal defense. This would explain how wild type *S*. Typhimurium colonizes these cells of the intestinal mucosa in wild type hosts [21], [53].

Apparently, greatly reduced virulence of *S*. Typhimurium is sufficient to cause enteropathy in *Cybb* mice. This is clearly not due to deficiency in immune regulation, because bacterial species not recognized as pathogenic are capable of triggering enteropathy in CGD. *S*. Tm^avir^ are a highly genetically amenable tool to study the mechanisms why. Direct and indirect susceptibility to ROS may be a determining feature of host microbiota species that permits their close relationship with the host.

## Materials and Methods

### Ethics Statement

All animal experiments and generation of new mouse-lines were approved by the legal authorities (licenses 201/2007 and 223/2010; Kantonales Veterinäramt Zürich, Switzerland) and carried out in the legally required manner.

### Mice

C57BL/6ptprc^b^ (congenic marker Ly5.2^+^; originally from Charles River), C57BL/6ptprc^a^ (congenic marker Ly5.1^+^; [61]), *Cybb* ^−/−^ (B6.129S-Cybb^tm1Din^/J; C57BL/6 background; [62]) and *Nos2* ^−/−^ (B6.129P2-Nos2^tm1Lau^/J; C57BL/6 background; [63]) were kept and bred under specific pathogen free (SPF) conditions. *Cybb* ^−/−/^ *Nos2* ^−/−^ mice have been described before and were generated by crossing *Cybb* ^−/−^ and *Nos2* ^−/−^ mice [13]. *Cybb* ^−/−/^DTR^+^ were generated by crossing *Cybb* ^−/−^ with DTR^+^ (B6.FVB-Tg[Itgax-DTR/EGFP]57Lan/J; [64]). *Cybb* ^−/−/^ *Myd88^−/−^* mice were generated by crossing *Cybb* ^−/−^ with *Myd88^−/−^* mice (C57BL/6 background; [65]). All newly generated double knockout mice and transgenic *Cybb* ^−/−^ mice bred and developed in a similar manner as *Cybb* ^−/−^ mice. All animals were kept under SPF conditions at the RCHCI of the ETH Zurich. For experiments mice were age (8–12 weeks old) matched and treated as described previously [19], [21]. In brief, mice were pretreated with streptomycin (1 dose, 25 mg/animal, by gavage). 24 h later mice were infected with 5×10^7^ cfu by gavage. Infections were performed for 12 h, 24 h, 72 h (3 days p.i.) and 96 h (4 days p.i.). Bacterial loads of gut lumen content, mLNs, livers and spleens were determined by plating [21].

### Generation of Mixed Bone Marrow Chimeras

The generation of bone marrow chimeras has been described before [21], [66]. Shortly, from euthanatized donor mice bone marrow from femur, tibia, brachium and pelvis was extracted. Recipient mice (*Cybb* ^−/−^) were γ-irradiated (1000 rad) and reconstituted with 2.5×10^6^ *Cybb* ^−/−^ (congenic marker Ly5.2) and 2.5×10^6^ C57BL/6ptprc^a^ (congenic marker Ly5.1) bone marrow cells intravenously. Animals were checked regularly and received drinking water containing Borgal© (Intervet) for 2 weeks. After 8 weeks, reconstitution efficiency was controlled after infection by flow cytometry (Ly5.1/CD45.1, Ly5.2/CD45.2) on LP cells. The reconstitution lead to a proportion of 69±3% *Cybb* ^−/−^ (Ly5.2) and 31±3% C57BL/6ptprc^a^ (Ly5.1) cells (analyzed: percentage of CD45.2 vs CD45.1 in the cecal LP, mean ± standard deviation).

### Bacterial Strains

*S*.Tm^avir^ (Δ*invG*; *sseD::aphT*; M557; [20]) and *S*.Tm*^invG^* (Δ*invG*; SB161; [67]) are isogenic derivatives of the wild type *Salmonella* SL1344 (*S*.Tm^wt^; [68]). For infection, bacteria were cultured in 0.3 M NaCl LB for 12 h at 37°C and subcultivated for 4 h as described before [69]. For detection of bacteria within mucosal tissue, bacteria harbored the reporter plasmid pM973 (*ssaH* promoter fused to *gfp*; [20]) or pM2121 (*ssaH* promoter fused to *mcherry*; this study).

### Mucosal Tissue Colonization and Cell-type Localization

Bacteria harboured a reporter plasmid expressing either *gfp* (pM973; [20]) or *mcherry* under the control of the *ssaH* promoter (pM2121; this study). For the evaluation of cecum-tissue invaded bacteria, the cecum tissue was fixed in 4% PFA and stored as described before [21]. 20 µm cryosections were stained with Armenian hamster anti-ICAM-I/CD54 (clone 3E2, 1∶100; Becton Dickson), DAPI (1∶1000, Sigma-Aldrich), Cy3-conjugated or Cy5-conjugated or FITC-conjugated goat anti-Armenian hamster IgG (1∶100, Jackson ImmunoResearch Laboratories) and AlexaFluor647 conjugated phalloidin (1∶100, Molecular Probes) [21], [66]. The average number of invaded bacteria in the epithelium and LP was evaluated by analyzing 3–9 tissue sections per mouse.

### Flow Cytometry

Cecum and mLNs were chopped and digested in RPMI (Invitrogen) and Liberase TL (Roche) for 45 min at 37°C under vigorous shaking. The resulting cell suspension was filtered through a 100 µm nylon cell-strainer (Milian) and stained in buffer containing PBS, 5 mM EDTA, 10% FCS and 50 µg/ml streptomycin. All fluorophore-labeled monoclonal antibodies were purchased from BD Biosciences or Biolegend. The LP cells were analyzed on a LSR II cytometer (Becton Dickinson) and graphs were produced with FlowJo software (Tree Star, Inc.).

### *In vivo* Dendritic Cell Depletion

DTX was injected i.p. (100 ng/25 g body weight; [64]) at 18 h before and 30 h after the infection. The depletion efficiency (>80%) and its negligible effect on other mucosal cell populations have been described before [21].

### Histopathological Evaluation

Tissues were embedded in OCT (Sakura, Torrance, CA) and snap-frozen in liquid nitrogen. Five µm cryosections were stained with hematoxylin and eosin (H&E). The degree of cecal mucosal tissue inflammation, i.e. edema, PMN infiltration, reduced numbers of goblet cells containing visible mucus-filled vacuoles and epithelium disruption, was judged by a pathologist yielding to a score of inflammation between 0–13 points as described before [19], [66].

### RT-qPCR

The excised cecum tissue was washed in cold PBS, placed in 600 µl RNAlater (Qiagen) and subsequently frozen at −80°C. Total RNA extraction was done using the RNeasy mini kit (Qiagen) with RNase-free DNase digest (Qiagen). For reverse-transcription of 1 µg mRNA aliquots, the RT^2^ HT First Strand cDNA Kit (Qiagen) was used. Custom RT^2^ Profiler PCR Arrays (Qiagen) were run with RT^2^ SYBR Green ROX FAST (QIAGEN) on an Applied Biosystems 7900 HT Fast Real-Time PCR System to amplify the resulting cDNA. Relative mRNA levels (2^−ΔCq^) were determined by comparing the PCR quantification cycle (Cq, determined with the Software SDS 2.2.1) for 27 genes related to inflammation and defense against *S*. Typhimurium infection (the selection is based on Songhet et al., 2010) with the reference gene *Actb*. The differences in their Cq cycles were calculated (ΔCq). In all experiments, the upper limit of Cq was fixed to 35 cycles. Then, the fold-increase over streptomycin-treated C57BL/6 mice was calculated and plotted. Each sample was controlled for mouse genomic DNA contamination. All DNA-positive data were excluded from further analysis. Lastly, RNA quality was monitored with the Agilent RNA 6000 Nano Kit (Agilent Technologies) on a 2100 Bioanalyzer (Agilent Technologies) and only samples with a RNA integrity number (RIN) >9.90 were included.

### Statistical Analysis

Statistical analysis was performed using the exact Mann-Whitney U test with the software GraphPad Prism 6. Values of p<0.05 (two tailed) were considered as significantly different between two groups. The minimal detectable bacterial colonization levels were set to 10 cfu/mLNs, 20 cfu/spleen, 60 cfu/liver (Fig. 1–4) or 30 cfu/liver (Fig. S4) or 10 cfu/g cecum content in cases where no bacteria were detected by plating. Messenger RNA levels of two groups were compared using Mann-Whitney U tests with Hochberg corrections for multiple comparisons using R x64 3.0.1 (Fig. 4F, S4G).

## Supporting Information

Figure S1 **NADPH oxidase is expressed in the infected mucosa and PMNs increase in number by infection.** C57BL/6 mice were pretreated with streptomycin and infected with *S*.Tm^wt^ for 12 h or 24 h, as indicated. RT-qPCR for *Cybb* expression in cecal tissues (A). Representative H&E sections (contrast and brightness were adjusted, color was enhanced, scale bar: 50 µm, arrow indicates a PMN) (B). Quantity of PMNs/high-power field (C). FC of cecal LP (pregated on CD45^+^ cells) (D). *: p<0.05; ns: not significant; red line: median; dashed line: detection limit.
(TIF)

Figure S2 ***Cybb*** **(but not iNOS) is important in mucosal defense against** ***S*** **.Tm^avir^ infection.** C57BL/6 mice (data replotted from Fig. 1), *Nos2* ^−/−^ mice (C57BL/6 background), *Cybb* ^−/−^ *Nos2* ^−/−^ mice (C57BL/6 background) or *Cybb* ^−/−^ mice (C57BL/6 background; data replotted from Fig. 1) were pretreated with streptomycin and infected for 4 days with *S*.Tm^avir^. The bacterial loads in the gut lumen (A), the LP (red (B)) or the epithelial cells of the cecum (blue (B)), the degree of mucosal inflammation (C) and bacterial loads in the mLNs (D), livers (E) and spleens (F) were analyzed. *: p<0.05; **: p<0.01; ns: not significant; red line: median; dashed line: minimal detectable value.
(TIF)

Figure S3 **Immunohistology of S.TminvG infected wild type C57BL/6 mice and S.Tmavir infected Cybb^−/−^** **mice is similar.** Cryo-sections of the cecal tissue from streptomycin pretreated wild type and *Cybb* ^−/−^ mice infected for 3 days with *S*.Tm*^invG^* or for 4 days with *S*.Tm^avir^, were stained with antibodies against CD11c (A), CD11b (B), CD68 (C), Gr-1 (D), CD3 (E) and CD8 (F) and imaged by bright field microscopy. The different times of infection are explained by the different disease kinetics of *S*.Tm*^invG^* and *S*.Tm^avir^. The former requires 3 days (in C57BL/6 mice) and the latter 4 days (in *Cybb* ^−/−^ mice) before overt inflammation of the cecal tissue is observed. The left panel shows representative pictures. The right panel shows the quantification. *: p<0.05; **: p<0.01; ns: not significant. Data is displayed as mean + SEM. *S*.Tm*^invG^* was able to elicit gut inflammation in wild type C57BL/6 and in *Cybb* ^−/−^ mice. In contrast, *S*.Tm^avir^ triggered enteropathy only in the *Cybb* ^−/−^ mice, but not in wild type C57BL/6 animals. Please note that the inflammatory lesions in the *S*.Tm^avir^ infected *Cybb* ^−/−^ mice displayed localized inflammatory lesions of equivalent immuno-histopathology as the lesion triggered by *S*.Tm*^invG^* in C57BL/6 mice.
(TIF)

Figure S4 ***S*** **.Tm** ***^invG^*** **infection in wild type C57BL/6 mice and** ***S*** **.Tm^avir^ infection in** ***Cybb*** **^−/−^ mice are similar.** C57BL/6 mice were pretreated with streptomycin and infected with *S*.Tm*^invG^* for 3 days. *Cybb* ^−/−^ mice were pretreated with streptomycin and infected with *S*.Tm^avir^ for 4 days. The bacterial loads in the gut lumen (A), the degree of mucosal inflammation (B), representative H&E pictures (contrast and brightness were adjusted and color was enhanced, scale bar: 200 µm, C) and bacterial loads in the mLNs (D), livers (E) and spleens (F) were analyzed. *: p<0.05; **: p<0.01; ns: not significant; red line: median; dashed line: minimal detectable value. Relative mRNA expression levels were compared between *S*.Tm*^invG^* infected C57BL/6 mice and *S*.Tm^avir^ infected *Cybb* ^−/−^ mice, data replotted partly in Figure 4 (G). Data is displayed as mean + SEM, differences were not significant (G).
(TIF)
