# Supplementary figures and images for: NADPH Oxidase Deficient Mice Develop Colitis and Bacteremia upon Infection with Normally Avirulent, TTSS-1- and TTSS-2-Deficient Salmonella Typhimurium
Source: PLoS One. 2013 Oct 15;8(10):e77204. doi: 10.1371/journal.pone.0077204 (PMC3797104; doi:10.1371/journal.pone.0077204)

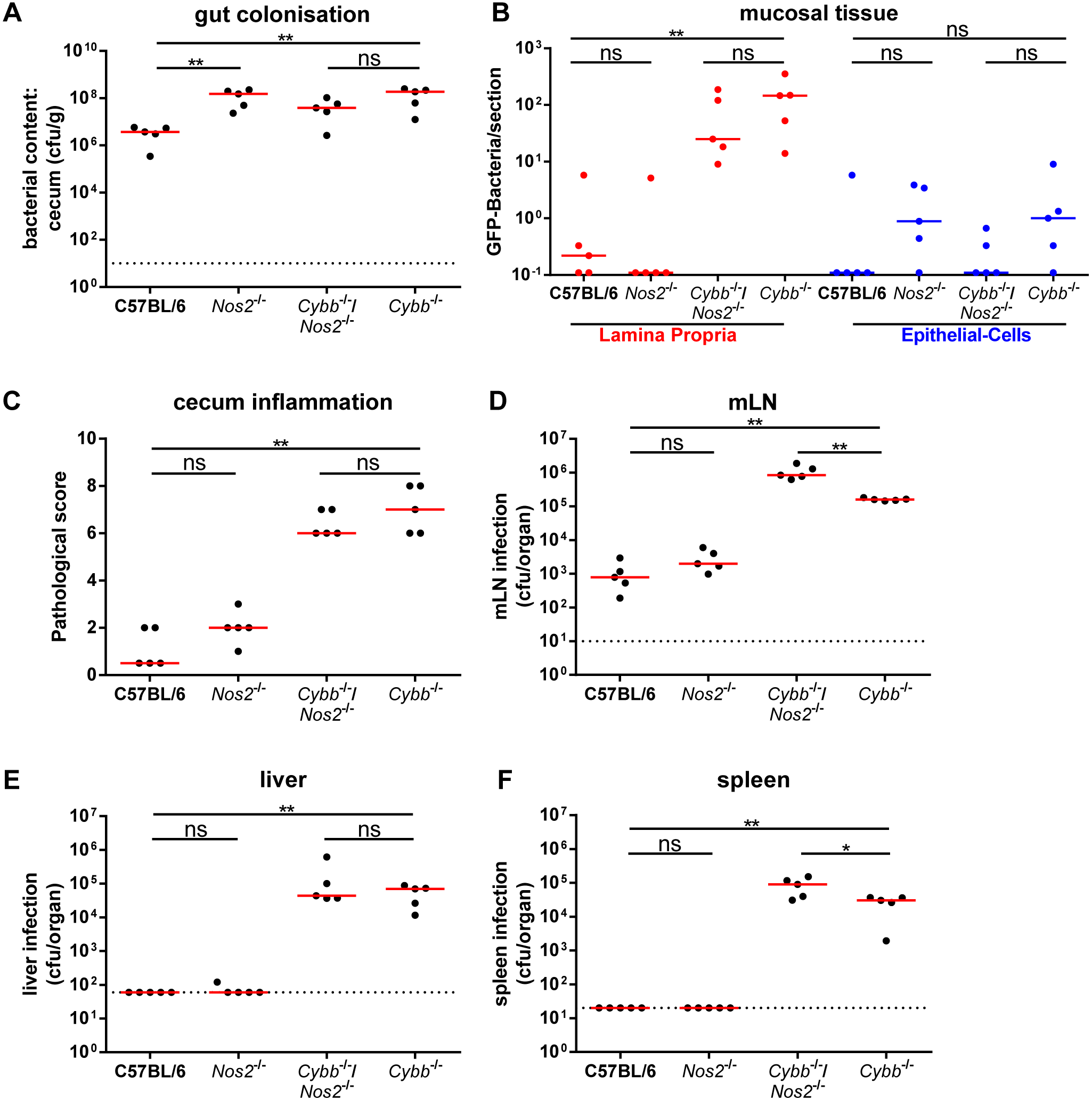

Supplement: Figure S1 — NADPH oxidase is expressed in the infected mucosa and PMNs increase in number by infection. C57BL/6 mice were pretreated with streptomycin and infected with S.Tmwt for 12 h or 24 h, as indicated. RT-qPCR for Cybb expression in cecal tissues (A). Representative H&E sections (contrast and brightness were adjusted, color was enhanced, scale bar: 50 µm, arrow indicates a PMN) (B). Quantity of PMNs/high-power field (C). FC of cecal LP (pregated on CD45+ cells) (D). *: p<0.05; ns: not significant; red line: median; dashed line: detection limit. (TIF) [file pone.0077204.s001.tif]

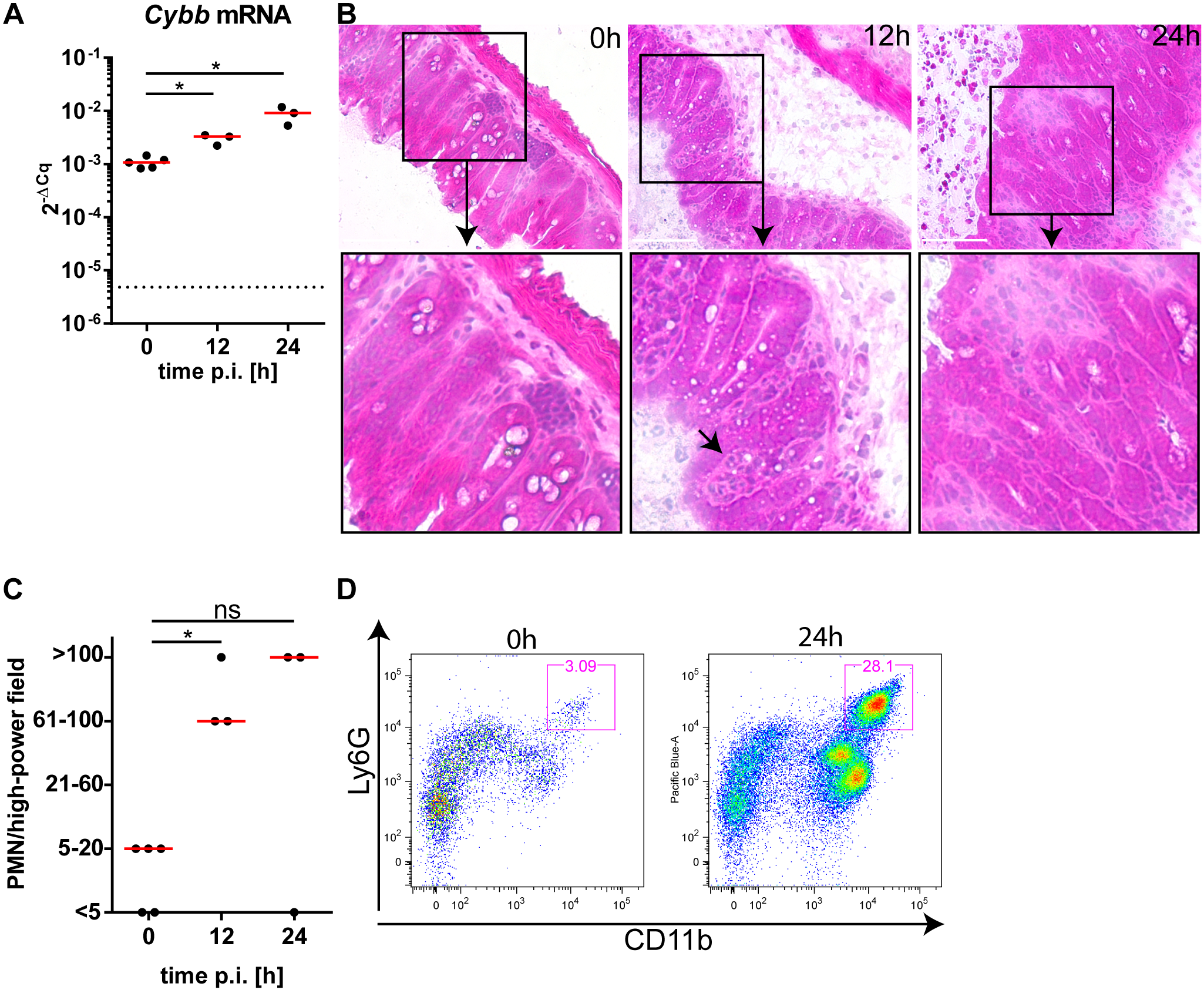

Supplement: Figure S2 — Cybb (but not iNOS) is important in mucosal defense against S .Tmavir infection. C57BL/6 mice (data replotted from Fig. 1), Nos2 −/− mice (C57BL/6 background), Cybb −/− Nos2 −/− mice (C57BL/6 background) or Cybb −/− mice (C57BL/6 background; data replotted from Fig. 1) were pretreated with streptomycin and infected for 4 days with S.Tmavir. The bacterial loads in the gut lumen (A), the LP (red (B)) or the epithelial cells of the cecum (blue (B)), the degree of mucosal inflammation (C) and bacterial loads in the mLNs (D), livers (E) and spleens (F) were analyzed. *: p<0.05; **: p<0.01; ns: not significant; red line: median; dashed line: minimal detectable value. (TIF) [file pone.0077204.s002.tif]

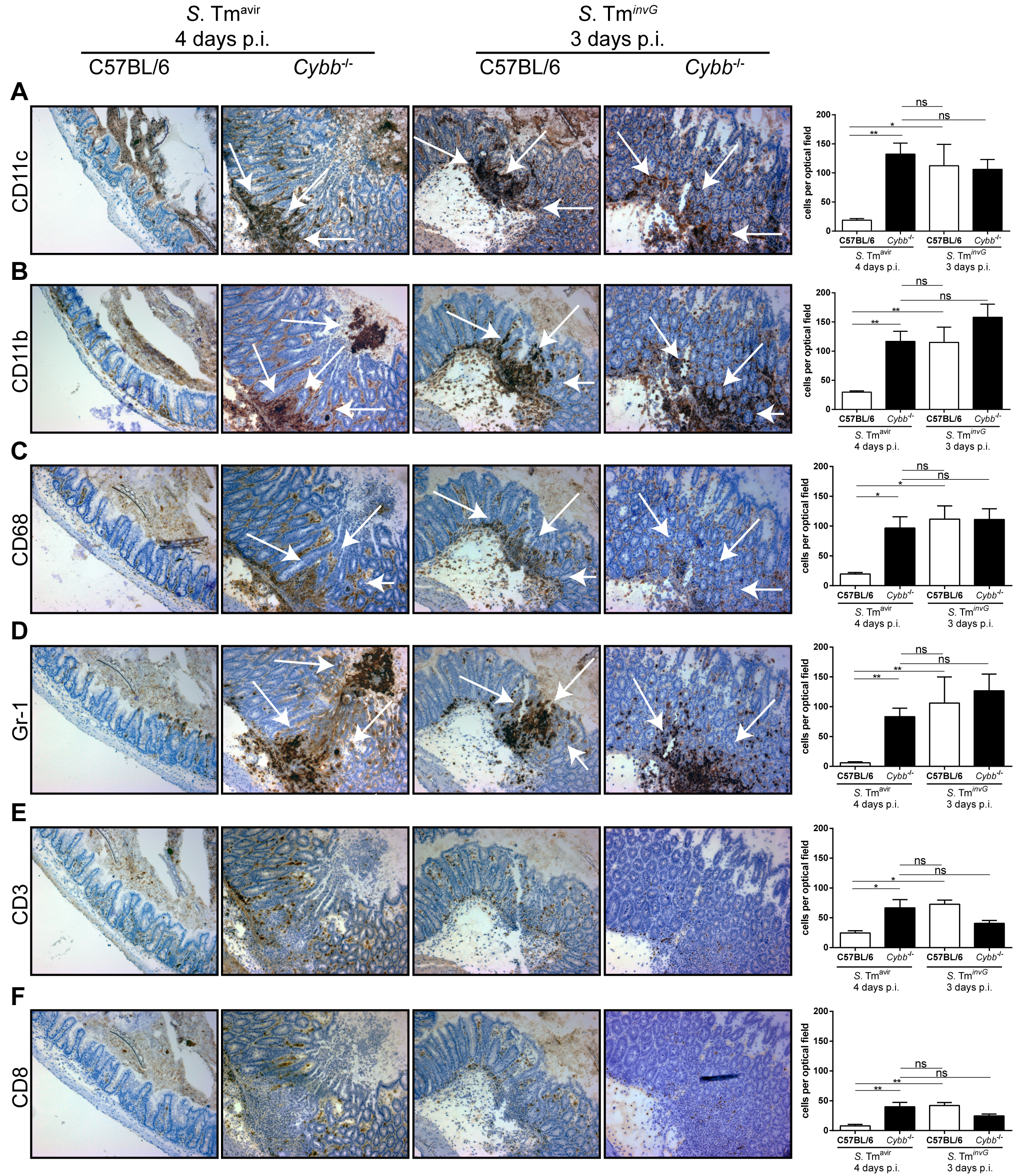

Supplement: Figure S3 — Immunohistology of S.TminvG infected wild type C57BL/6 mice and S.Tmavir infected Cybb−/− mice is similar. Cryo-sections of the cecal tissue from streptomycin pretreated wild type and Cybb −/− mice infected for 3 days with S.TminvG or for 4 days with S.Tmavir, were stained with antibodies against CD11c (A), CD11b (B), CD68 (C), Gr-1 (D), CD3 (E) and CD8 (F) and imaged by bright field microscopy. The different times of infection are explained by the different disease kinetics of S.TminvG and S.Tmavir. The former requires 3 days (in C57BL/6 mice) and the latter 4 days (in Cybb −/− mice) before overt inflammation of the cecal tissue is observed. The left panel shows representative pictures. The right panel shows the quantification. *: p<0.05; **: p<0.01; ns: not significant. Data is displayed as mean + SEM. S.TminvG was able to elicit gut inflammation in wild type C57BL/6 and in Cybb −/− mice. In contrast, S.Tmavir triggered enteropathy only in the Cybb −/− mice, but not in wild type C57BL/6 animals. Please note that the inflammatory lesions in the S.Tmavir infected Cybb −/− mice displayed localized inflammatory lesions of equivalent immuno-histopathology as the lesion triggered by S.TminvG in C57BL/6 mice. (TIF) [file pone.0077204.s003.tif]

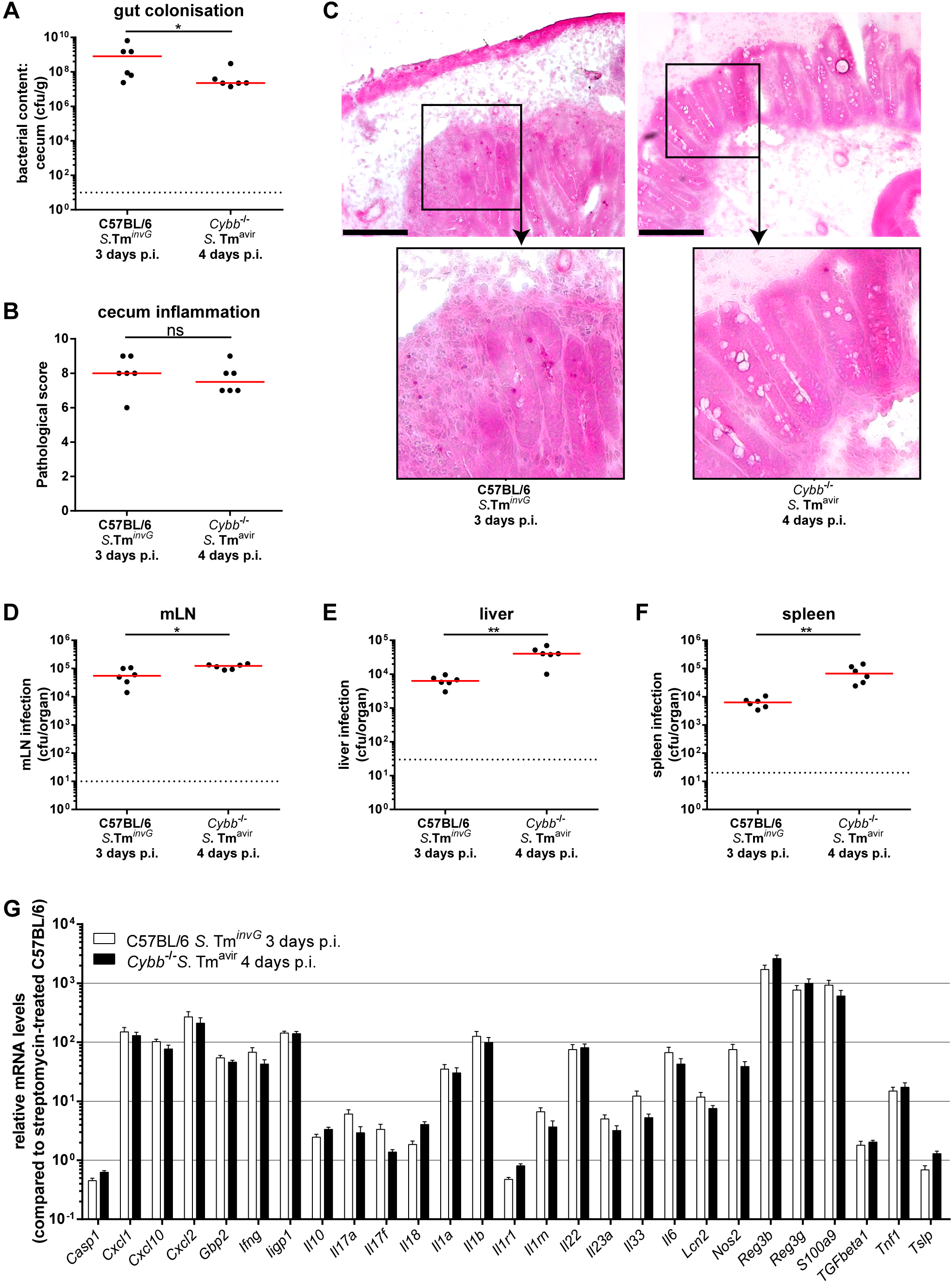

Supplement: Figure S4 — S .Tm invG infection in wild type C57BL/6 mice and S .Tmavir infection in Cybb −/− mice are similar. C57BL/6 mice were pretreated with streptomycin and infected with S.TminvG for 3 days. Cybb −/− mice were pretreated with streptomycin and infected with S.Tmavir for 4 days. The bacterial loads in the gut lumen (A), the degree of mucosal inflammation (B), representative H&E pictures (contrast and brightness were adjusted and color was enhanced, scale bar: 200 µm, C) and bacterial loads in the mLNs (D), livers (E) and spleens (F) were analyzed. *: p<0.05; **: p<0.01; ns: not significant; red line: median; dashed line: minimal detectable value. Relative mRNA expression levels were compared between S.TminvG infected C57BL/6 mice and S.Tmavir infected Cybb −/− mice, data replotted partly in Figure 4 (G). Data is displayed as mean + SEM, differences were not significant (G). (TIF) [file pone.0077204.s004.tif]
